# Return to Work: A Cut-Off of FIM Gain with Montebello Rehabilitation Factor Score in Order to Identify Predictive Factors in Subjects with Acquired Brain Injury

**DOI:** 10.1371/journal.pone.0165165

**Published:** 2016-10-25

**Authors:** Marco Franceschini, Maria Pia Massimiani, Stefano Paravati, Maurizio Agosti

**Affiliations:** 1 IRCCS San Raffaele Pisana, Rome, Italy; 2 San Raffaele University, Rome, Italy; 3 San Raffaele Portuense, Rome, Italy; 4 Department of Physical Medicine and Rehabilitation, Tor Vergata University, Rome, Italy; 5 University-Hospital of Parma, Parma, Italy; University of South Florida, UNITED STATES

## Abstract

Return to work (RTW) for people with acquired brain injury (ABI) represents a main objective of rehabilitation: this work presents a strong correlation between personal well-being and quality of life. The aim of this study is to investigate the prognostic factors that can predict RTW after ABI (traumatic or non- traumatic aetiology) in patients without disorders of consciousness (e.g. coma, vegetative or minimally conscious state) at the beginning of their admission to rehabilitation. At the end of a 6-month follow-up after discharge, data were successfully collected in 69 patients. The rehabilitation effectiveness (functional Recovery) between admission and discharge was assessed by Functional Independent Measure (FIM) gain, through the Montebello Rehabilitation Factor Score (MRFS), which was obtained as follows: (discharge FIM—admission FIM)/(Maximum possible FIM—Admission FIM) x 100. The cut-off value (criterion) deriving from MRFS, which helped identify RTW patients, resulted in .659 (sn 88.9%; sp 52.4%). Considering the Mini Mental State Examination (MMSE) and the MRFS data, the multivariable binary logistic regression analysis presented 62.96% of correct RTW classification cases, 80.95% of non-RTW leading to an overall satisfactory predictability of 73.91%. The results of the present study suggest that occupational therapy intervention could modify cut-off in patients with an MFRS close to target at the end of an in-hospital rehabilitative program thus developing their capabilities and consequently surpassing cut-off itself.

## Introduction

In recent years, the gradual improvement of assistance during emergencies and intensive care has led to an increase in the number of people worldwide who survive after having acquired brain injury (ABI). ABI includes non-traumatic aetiology, such as cerebrovascular diseases (75%) and traumatic aetiology (25%) [1]. At the end of rehabilitation, a large number of these people experience considerable limitations to their activities, interfering with their quality of life and productivity and representing an economic hardship for society [2]. Specifically, ABI-related disability increases both direct and indirect social costs, connected with the need for long-term assistance and loss of productivity of the person involved and, in some cases, of the entire family unit [3, 4]. This is most obvious and tragic when people with ABI are young, with a long life expectancy and prospects of going back to their previous jobs. Return to work (RTW)is one of the main objectives of rehabilitation projects carried out by multi-professional team sand is often used as an end point to evaluate the true effectiveness of all rehabilitation activities. In fact, work is one of the most important targets that allow us to measure the real participation level of working-age people: it is well known that working activity is strongly correlated with a better sense of well-being and quality of life [5, 6, 7, 8].

Literature has not yet clarified the percentage of RTW subsequent to ABI. Studies show sizeable differences, from 13% to 73%, depending on the lesion severity of the people involved in the studies [9, 10]. According to another author, a patient with moderate ABI is able to RTW within 2 years in.40% of cases [11]. In such cases, failure to RTW may result in a considerable emotional impact on patients and caregivers with important psychological, social and economic consequences. In particular RTW should be considered one of the main goals of the rehabilitative process in subjects who have experienced ABI. In conclusion, work is an essential part of daily life that can modify social integration, participation and expectancy and quality of life [6]. The objective of this study is to identify the key-factors which can predict RTW after ABI and determine whether these factors can be managed so as to increase chances for employment in patients with residual disability.

## Materials and Methods

### Participants, timing and assessment tools

Patients diagnosed with an acquired brain injury were enrolled over a 2-year period from inpatients consecutively admitted to the Rehabilitation Unit of our Clinic. Informed consent was collected from all patients. The inclusion criteria were: ABI, age between 18 and 65 years and being employed before their acute brain injury episode. The exclusion criteria were: suspected or known diagnosis of inflammatory or degenerative progressive disease (i.e. multiple sclerosis, Parkinson’s or Alzheimer’s disease) or the presence of consciousness disorders such as coma, vegetative or minimally conscious state.

Patients were evaluated at admission (T0), at discharge (T1) and after 6 months (T2). Assessment tools included the Cumulative Illness Rating Scale (CIRS), the Mini–Mental State Examination (MMSE), the Functional Independence Measure (FIM), the Trunk Control Test (TCT), the Rankin Scale and the Motricity Index (MI). The effectiveness of functional recovery as a result of the rehabilitation treatment was also assessed through the Montebello Rehabilitation Factor Score (MRFS), derived from the FIM and obtained as follows: (discharge FIM—admission FIM)/(Maximum possible FIM—Admission FIM**)** x 100 [12, 13, 14]. The main outcome was RTW, intended both as a full or part time job.

In addition, two differently structured interviews, consisting of a specific questionnaire, were administered to the recruited patients. The first questionnaire, administered at T0, explored demographic, social and previous employment characteristics of patients. The second one was administered at T1 and was used to collect expectations for future employability. All patients were evaluated by experienced examiners previously trained to use the above assessment tools (see S1 Appendix).

The study was approved by the Ethics Committee (“Comitato Etico IRCCS San Raffaele Pisana”, Via di Val Cannuta, 247–00166 Rome), on November 29th 2010 (protocol # 11/2010). In order to take part in this study, each participant provided written informed consent.

### Statistical analysis

Data were presented as mean and medians (min, max). Group differences in demographic and clinical data were assessed using parametric (Student’s test or ANOVA) and non-parametric (χ^2^ and Mann-Whitney) tests. Multivariate analyses were performed using logistic binary regression models in order to identify multiple relations between a variable of interest and two or more explicative variables. Inclusion of explicative variables in the models followed stepwise procedures (forward and backward), with specific motivations for each variable. Where appropriate, the individual variables included were reported with their Odds Ratio, and the significance of each coefficient in the model was examined. Non-significant variables with a p-value greater than 0.05 were removed from the model in a step-by-step process, starting with the variables showing the highest probability levels. Each time a variable was excluded, the integrity of the model was checked with the Hosmer-Lemeshow test.

**Fig 1:**
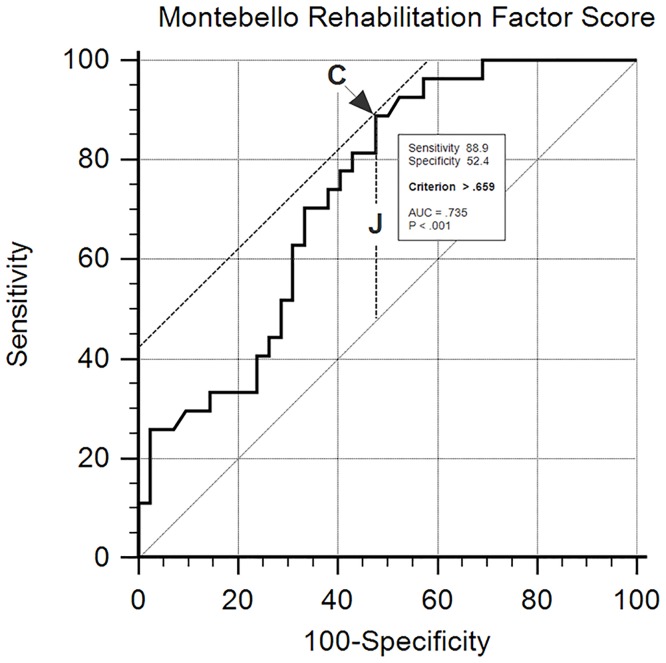
Model to identify a cut-off value of Montebello Rehabilitation Factor Score (MRFS). C is the criterion, J = sensitivity (C) + specificity (C). J finding the best cut-off point that is equivalent to measuring the J of Youden Index. Youden Index is the greatest vertical distance between ROC curve and the diagonal line.

Once a predictive model of RTW had been defined, it was investigated whether there was a cut-off point of the MRFS (the independent variable) that could predict the *employment status* category (employed or unemployed) of each subject. Where a reference criterion was available, receiver-operating characteristic (ROC) analyses offered an elaborate method for constructing cut-off points [15]. Having used a continuous variable such as MRFS, in which the sensitivity and specificity have the same statistical weight, the best cut-off point for obtaining a positive result from the test was the maximum value which could be obtained for both of these aspects in which the sum was the highest possible. This was necessary in order to identify patients unable to “RTW”. With this procedure, the determination of the cut-off point coincided with the achievement of the minimum value of false negative and false positive, which depends on classification errors. The cut-off point obtained with this method has the characteristic of reaching the best-expected objective, that is to say: maximize the potential for correct diagnosis and minimize classification errors. In the case in which “c” is the best cut-off point of the test results, Youden introduced the following index for the ROC curve: J = sensitivity (c) + specificity (c). Moreover, finding the best cut-off point is equivalent to measuring the “J” of the Youden Index. This index is an important synthesis of the ROC curve. From a graphical point of view, the Youden Index is the greatest vertical distance between the ROC curve and the diagonal line (Fig 1). Said graph presents a complete optimal potential measurement of the diagnostic capacity regarding clinical activity. ROCs describe the relation between sensitivity and specificity for different cut-off points. ROC analyses provide an evaluation of the ability diagnostic instruments have to discriminate between health and disease.

The choice of cut-off points requires a trade-off between:

1. High sensitivity, which means the likelihood of identifying an actual risk (i.e., "*employment status—*RTW") through a positive test result.
2. High specificity, which means the likelihood of identifying a non-existent risk (i.e., "*employment status—*not RTW") through a negative test result.

Assuming that sensitivity and specificity are of equal importance, the maximum of the Youden Index indicates an optimal cut-off point [16]. The overall ability of a measure to discriminate between healthy and diseased subjects is indicated by the magnitude of the area under the curve (AUC). We know that a correlation exists between the positive predictive value (PPV) and the negative predictive value (NPV), and that the prevalence, which in our sample refers to people able to achieve “*employment status—*RTW” in any case, is unknown. It is also noted that if the prevalence of the disease in the population is high, the results of all the tests are reliable but, in this case, we do not know the real prevalence of people that RTW [17, 18].

The software packages “IBM SPSS version 22.0” and “MedCalc version 16.1.0” were used for analyses.

## Results

### Participants

**Fig 2:**
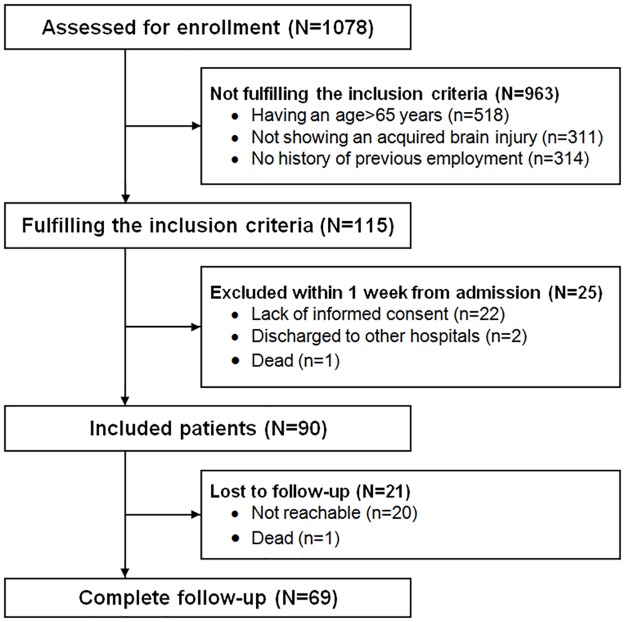
Flow chart of patients who met inclusion/exclusion criteria for the study.

Out of 1,078 patients admitted to the Rehabilitation Unit, 963 did not meet the inclusion criteria, mainly because of the age (n = 518), secondly because they were not experiencing ABI (n = 311) and in the last place because they did not have a history of previous employment (n = 134). 115 patients fulfilled the inclusion criteria. Of these, 25 were excluded either for not giving the informed consent (n = 22) or because they were discharged early to other hospitals (n = 2) or owing to death (n = 1) within one week of admission. A total of 90 patients were included in the protocol. At the end of the study, data were successfully collected from 69 patients while 21 were lost at the follow-up as no-shows (n = 20) or owing to death (n = 1) (Fig 2). An analysis of the main variables of subjects lost at follow-up has shown how they did not differ from the rest of the study population at any variable, with the exception of their mean age (younger, p = .026). All the subjects included were right-handed. The most frequent ABI disease was stroke (n = 55; 79.7%), followed by traumatic brain injury (n = 6; 8.7%), and benign brain tumour (n = 8; 11.6%). Demographic and clinical characteristics of investigated patients are reported in Table 1.

**Table 1:** Demographic and clinical characteristics at baseline.

Characteristic	All	Return to Work		P-value
		No	Yes	
		n (%) Median [25^th^-75^th^ PR]		
Subject, n	69	42	27	
Age, years	56.0 [50.5–62.0]	56.0 [50.5–59.6]	56.0 [52.0–62.0]	.442^a^
Gender, male	55 (79.7)	36 (85.0)	19 (70.0)	.122^b^
*Index event*				.631^b^
Stroke	55 (79.7)	32 (76.2)	23 (85.2)	
Traumatic brain injury	6 (8.7)	4 (9.5)	2 (7.4)	
Benign brain tumour	8 (11.6)	6 (14.3)	2 (7.4)	
*Hemispheric involvement*				.069^b^
Unilateral, right	42 (60.9)	21 (50.0)	21 (77.8)	
Unilateral, left	23 (33.3)	18 (42.9)	5 (18.5)	
Bilateral	4 (5.8)	3 (7.1)	1 (3.7)	
MMSE	26.0 [24.0–27.3]	25 [23.0–26.0]	27.0 [25.0–28.0]	< .004 ^a^
TCT	51.0 [24.0–100.0]	48 [12.0–77.2]	61.0 [48.0–100.0]	.040 ^a^
MI, right	80.0 [64.5–100.0]	76.5 [61.3–100.0]	88.0 [76.5–100.0]	.039 ^a^
MI, left	70.5 [36.5–100.0]	60.2 [32.8–100.0]	76.5 [54.5–100.0]	.327 ^a^
CIRS	21.0 [19.0–22.5]	21.0 [20.0–23.0]	21.0 [19.0–22.0]	.199 ^a^
FIM	65.0 [48.0–76.5]	53 [46.0–69.2]	75.0 [59.0–87.0]	< .0001^a^
Rankin	4.0 [3.0–4.0]	4.0 [4.0–4.0]	4.0 [3.0–4.0]	.089 ^a^

^a^ Mann-Whitney test.

^b^ Chi-Square test.

*Abbreviations*: *SD*, Standard Deviations; *PR*, Percentile Range; *MMSE*, Mini Mental State Examination; *TCT*, Trunk Control Test; *MI*, Motricity Index; *CIRS*, Cumulative Illness Rating Scale; *FIM*, Functional Independent Measure.

### Six-month employment status

Among investigated patients, 42 (60.9%; 36 men and 6 women, mean age 56.0) had not returned to work at the 6-month follow-up, while 27 (39.1%; 19 men and 8 women, mean age 56.0) were employed again. 23 patients (85.2%) resumed their previous employment status, while 4 (14.8%) changed job type, job timetable or other job-related characteristics. Higher rates of RTW were found in patients permanently employed (n = 22, 81.5%) before the injury as opposed to patients without previous permanent employment (n = 5, 18.5%). Even patients who had previously held a manual post (n = 19, 70.4%) showed a greater rate of RTW compared to patients previously employed in mental work (n = 8, 29.6%).

### Predictors of post-injury employment

Age, gender, aetiology of ABI and side of paresis showed no significant differences in the two groups (RTW and non-RTW).

MMSE and TCT highlighted a significantly better performance on the tests in people who RTW, while MI showed a statistical difference in favour of people who RTW with only a healthy left hemisphere. The comorbidities (CIRS) and disability/participation (Rankin) had no bearing on RTW prediction.

Strong significance instead emerged from the activities evaluation, which was assessed using the FIM (Table 1). The MRFS evaluated at T1 and at T0 as a relative functional gain showed a significant prediction in favour of RTW (.820 [.679-.906] vs. .652 [.380-.851] p <0001).

The predictors of post-injury employment collected in our questionnaire (Table 2) only showed a significant influence on RTW rates for freelance or independent work (p = .043) or on the potential of reintegration in the previous job (p = .011).

**Table 2:** Predictors of post-injury employment.

Characteristic	All	Return to Work		P-value^a^
		No	Yes	
Subject, n	69	42	27	
***Characteristic at T0***, *yes/no*				
Living alone	26%	21%	33%	
Barriers at home	13%	7%	22%	
Barriers in the neighbourhood	16%	21%	11%	
Barriers at work	14%	14%	15%	
Public transport	32%	33%	30%	
Driver's license	84%	86%	81%	
High school and University	43%	45%	41%	
Manual work	67%	67%	70%	
Self-employment (freelance work or Independent work)	30%	21%	44%	.043
***Characteristic at T1***, *yes/no*				
Availability job placement (job integration)	74% ^b^	67%	85%	
Potential for reintegration in the job previously held	70% ^b^	58%	89%	.011
Potential for reintegration in a job other than the one previously held	31% ^b^	33%	28%	
Potential for vocational training	22% ^b^	21%	24%	

^a^ Chi-Square and Exact test (reported as significant).

^b^ Some people did not answer this question.

**Fig 3:**
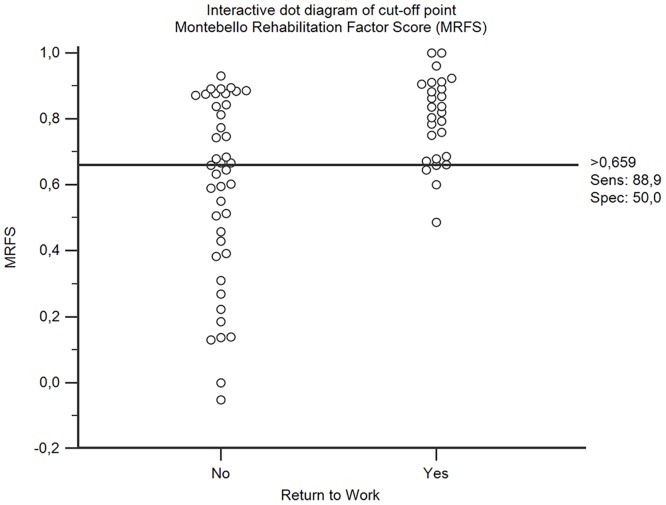
Interactive dot diagram of cut-off point of the Montebello Rehabilitation Factor Score (MRFS is relative functional gain) for "Return to Work".

On the basis of the multivariate analysis, the MMSE score (OR 1.301; 95% CI 1.020–1.660) and the MRFS (OR 109.396; 95% CI 3.723–3214.390) were independent predictors of employability. ROC analysis defined a cut-off (> .659) for the variable MFRS predictive for of the ability to RTW (Tables 3 and 4, Fig 3).

**Table 3:** RTW predictors according to Binary logistic regression.

	**Coefficients**	**OR (95% CI)**	**P-value**
**Intercept**	-10.570		
**MMSE** (for one point increase)	.263	1.301 (1.020–1.660)	.0341
**MRFS** (for one point increase)	4.695	109.396 (3.723–3214.390)	.0065
**Nagelkerke R**^**2**^ = .335			
**Area Under ROC curve** = .797, **95%CI** = .683–.884			
**Classification table** (cut-off value p = .50)			
**Actual Group**	**Predicted Group**		**Percent Correct**
	Return to Work (yes)	Return to Work (no)	
Return to Work (yes)	17	10	62.96%
Return to Work (no)	8	34	80.95%
Percent of cases correctly classified			73.91%

*Abbreviation*: *MMSE*, Mini Mental State Examination; *MRFS*, Montebello Rehabilitation Factor Score; *CI*, confidence interval.

**Table 4:** RTW predictors according to the ROC curve optimal criterion.

MRFS (cut-off)	SE (95% CI)	SP (95%CI)	+PV (95% CI)	-PV (95% CI)	AUC (95% CI)
>**.659**	.889 (.708–.976)	.524 (.364–.680)	.545 (.388–.696)	.880 (.688–.975)	.734 (.614–.833)

*Abbreviation*: *MRFS*, Montebello Rehabilitation Factor Score; *CI*, confidence interval; *SE*, sensitivity; *SP*, specificity; *+PV*, positive predictive value; *-PV*, negative predictive value; *AUC*, Area Under the ROC curve (maximum = 1.0)

## Discussion

The main object of this study was to identify key-factors, which alone or combined could predict the RTW after ABI, and determine whether these factors could be managed to improve the employment chances in patients with residual disability. Until now, few studies have compared the individual factors or the combinations of prognostic factors in order to highlight their relationship with RTW in patients with traumatic or non-traumatic ABI. In their review, Van Velzen and Colleagues described strong evidence of negative prognostic value for RTW in relation to the length of hospitalization after traumatic ABI. Anamnestic or mental state (depressive, anxious) variables did not, on the other hand, predict the return to work after non traumatic lesions. These authors found weak evidence of influence over RTW for ‘number of associated injuries’, ‘activities of daily living (ADL)’ and ‘residual physical deficits’. The authors concluded that these three aspects could improve after specific rehabilitative training, and that the low number of persons who RTW after ABI was surprising. The weakness of ADL assessment as a strong prognostic factor depends on the different systems used in the studies to evaluate patients [5].

As reported by various authors, a program focused on early intervention and early RTW is an effective way to reduce the rate of health-related absences and to shorten the period of time an employee is absent from work. If, on the one hand, work helps people to be healthy and feel well, not going to work, on the other hand, or developing a work disability as well as being unemployed undermines health and wellbeing. As reported by various authors, unemployment often results in lowered self-esteem and in the loss of social life and community role. [5, 6, 19].

Considering circumstances that increase or decrease, RTW may help ABI patients to go back to work earlier. ABI is one of the main causes of morbidity affecting cognitive, sensitive and motor functioning at the same time [20, 21]. Behavioural and psychiatric disorders frequently occur in subjects following an ABI [22]. Our findings showed and confirmed the importance of the cognitive status of subjects in the wake of ABI. Post-injury employability (RTW) is strongly influenced by cognitive performances, as evaluated by the MMSE. Other good predictors were the Trunk Control Test, the side of the lesion as well as the percentage of functional recovery following rehabilitation, as expressed by the MRFS.

Other interesting data emerged from this study. Above all, the conservation of motor skills of the right side of the body ensures a greater chance of RTW, presumably in relation to the retention of motor activity of the upper limb.

The Van Velzen [5] review underlines how few studies have investigated the role of external factors to predict RTW after ABI. Our questionnaire attempted to explore this area but only the characteristics of previous employment influenced RTW: patients who work for themselves are more likely to go back to work than those in permanent employment. Unlike what has been found in patients with spinal cord injury [23, 24], preserving the ability to drive was not an independent predictor of post-injury employment in our patients. This may be explained by taking into account the nature of the primitive disease and its functional consequences on ADL. In particular, patients with spinal cord injury are likely to have motor disability, non-independent mobility, in the absence of cognitive and neurophysiological impairment [23, 24]. Conversely, patients with moderate ABI may show a variable combination of symptoms in motor, sensory and cognitive domains, which may interfere with independence and employability, regardless of whether they have maintained the ability to drive.

In scientific literature this is the first paper to report a direct relationship between post-injury employability and the MRFS as a means for reporting the efficacy of rehabilitation programmes. In patients studied, the best combination of sensitivity and specificity of the MRFS as a predictor for RTW, has been found at a MRFS score cut-off >.659 (sensitivity 88.9; specificity 52.4), while prediction was less accurate for poor outcome MRFS (cut-off < .659), with the area under the curve at 73.5%. The more reduced specificity in respect to sensitivity is due to the fact that among those who do not RTW while having obtained a MRFS value > 0.659 still have a MMSE <24, which results in being one of the two predictors in multivariate analysis. In fact it is the cognitive impairment present at admission to hospital (as documented by the MMSE values) that hinders RTW despite adequate MRFS. This allows the identification of an MRFS cut-off to predict post-injury employability, the stratification of patients on the basis of their probability of being employed at discharge, and rehabilitative tailoring approaches to single out patients and their potential for recovery. During the final phase of rehabilitation, it can be extremely useful to persist with forms of occupational therapy to surpass cut-off with a better chance of RTW.Cifu et Al. in Traumatic Brain Injury found that at admission FIM had a significant influence on RTW, but not in FIM gain between admission and discharge [25]. The use of MRFS normalizes the data of gain regardless of the FIM value at admission, strengthening its relation with RTW.In order to guarantee the highest possibilities of RTW it could also be important to intensify cognitive rehabilitation activities.

Some authors have estimated high levels of direct and indirect costs after ABI [4, 5]. In their article TeAo et Al. concluded highlighting the importance of developing specific measures to prevent the risk of not returning to work (RTW) both in mild and moderate ABI, especially in respect of long term projections where the number of people who do not RTW after Traumatic Brain Injury is increasing considerably [26]. Early vocational rehabilitation is becoming an important practice which influences the RTW of people affected by ABI. The cut-off deriving from the MRFS we administered may help identify people close to the cut-off level who might prove to have a very good chance of RTW. Thus it will be possible to intensify and target many more types of intervention combined with occupational therapy to increase the chances for these subjects to RTW.

### Limitations of the study

The low number of subjects taken into account could be a limitation of this paper. However another study with a higher number of persons has been planned. With this new study it will be possible to to report sensitivity, specificity, positive and negative predict values among those who RTW stratified by MMSE.

The high rate of loss at follow-up (23.3%), is thought to be mainly due to the large area from where recruited patients come from and the related difficulties of their relatives who were to have brought them to the hospital for medical controls. As the further analysis of lost patients at follow-up shows how their data do not differ much from the rest of the study population, we are fully confident in the results of our study.

## Conclusions

Although many variables were investigated, strong evidence was only found with MRFS in people experiencing non-traumatic ABI. Since some reservation on the role played by MMSE and other variables on MRFS remain, a new study with a larger sample size, in a multicentre context, has been planned. We are expecting to then be able to identify other MRFS cut-offs related to the variables taken into account, and to provide a better understanding of the role of occupational and vocational rehabilitation in relation to RTW.

## Supporting Information

S1 Appendix Questionnaire. Admission and dismission structured interwiew.
(DOCX)

S1 Table Database of the study. (XLSX)
