# Supplementary material for: Return to Work: A Cut-Off of FIM Gain with Montebello Rehabilitation Factor Score in Order to Identify Predictive Factors in Subjects with Acquired Brain Injury
Source: PLoS One. 2016 Oct 25;11(10):e0165165. doi: 10.1371/journal.pone.0165165 (PMC5079591; doi:10.1371/journal.pone.0165165)
Supplement: S1 Appendix — Admission and dismission structured interwiew. (DOCX) [file pone.0165165.s001.docx]

**Appendix 1**

**Questionnaire**

**ADMISSION**

| QUESTION | | ANSWEAR | |
| --- | --- | --- | --- |
| 1 | Do you live alone? | Yes | No |
| 2 | Do you have architectural barriers at home? | Yes | No |
| 3 | Are there any architectural barriers in your neighborhood? | Yes | No |
| 4 | Are there any architectural barriers at your place of work? | Yes | No |
| 5 | Which do you use more often public or private transportation? | Public | Private (personal transport) |
| 6 | Do you have a driving license | Yes | No |
| 7 | What is your level of education? | High school or University | Primary School |
| 8 | How would you describe your work activity? | Manual | Intellectual |
| 9 | Are you? | Employed | Self-employed |

**DISMISSION**

| QUESTION | | ANSWEAR | | |
| --- | --- | --- | --- | --- |
| 1 | In future, do you think that you will be able to start working again? | Yes | No | I don’t know |
| 2 | In future, do you think that you will be able to do the same job as you used to ? | Yes | No | I don’t know |
| 3 | In future, do you think that you will be able to do a job that is different from the one you used to do? | Yes | No | I don’t know |
| 4 | In future, do you think that you will be able to do the same job as you used to ? | Yes | No | I don’t know |
